# In an Obstetrical Case, Should the Physician Take His Forceps with Him?

**Published:** 1871-09

**Authors:** J. C. McMechan

**Affiliations:** Cincinnati, O.


### In an Obstetrical Case, should the Physician take his Forceps with Him?

By J. C. McMechan, M.D , of Cincinnati, O.

" What gift has Providence bestowed on man that is so dear to him as his children?"—*Cicero.*

It is the grand desire of married people to have progeny. As women sigh so for children, it certainly becomes the duty of a physician to try and deliver them as safely as possible.

What has brought this matter so vividly before my mind is a case that occurred in my practice a few nights ago.

I had been engaged to wait on Mrs. M. G., in confinement, and about 3 o'clock A.M., was summoned to her bedside. She had been in labor five hours, and the membranes ruptured at two o'clock. The feet had presented, and the child was born as far as the knees. In a few minutes the child was born all but the head, and that remained in the pelvis. The usual manœuvers on such occasions were tried, but without avail. I told the woman's husband to run to my office and bring my obstetrical instruments as quickly as possible. The office was distant but a square; but what were my regrets on his just returning, to find the pulsations in the cord had ceased. The instruments were quickly applied and the child delivered. Had I taken the instruments with me, I doubt not but that the child could have been saved, for after the delivery of the body the pulsations in the cord were quite strong and natural, and only ceased after about five minutes. This is the usual time, most authors say, a child can exist in that position.

To make me regret the delivering of a dead child the more deeply, was the fact that the mother had lost her previous child, her first one, in confinement; her grief was almost inconsolable on learning that the child was not alive.

In another case, which occurred about a year ago, I was called to see a lady in confinement, and found the very same presentation as in the case above related. As the patient lived a distance from my office, I took the precaution of taking my obstetrical instruments with me. I was at the house but a short time when the child's body was born, but the head remained behind. Finding delivery in the usual way impossible, I rapidly applied the forceps and delivered; the child seemed dead, but in a few minutes it was resuscitated, and is now a large and healthy child. It was the first confinement the lady had had, and the child would certainly have been lost if I had not had the forceps in the house at the time.

A physician going to visit an obstetric case knows not what operation he will have to perform, or that he will find it necessary to interfere at all, but it is best to be prepared for emergencies. There is a great prejudice existing among the people against the use of instruments, and it is partly through fear of remarks that physicians are deterred from taking them along. Physicians, of course, are justified in being cautious and not getting the prejudices of people unnecessarily aroused, but if one child even in two hundred can be saved by their carrying a pair of forceps with them, they should do so.

About three months ago I was asked by a gentleman to see his wife, who had been in labor for a number of hours, and who had employed a midwife to wait upon her. It being about one o'clock at night, and supposing the case might be a difficult one, I took my forceps with me. On arriving at the house, I left them in an ante-room, and then examined the patient. Everything being favorable, and not requiring interference, I left, saying I would return early in the morning. I told the woman's husband that I thought his wife would be delivered naturally in a few hours, that I would leave the case in the hands of the midwife, and if she was not delivered by morning I would use the instruments.

On going back in the morning, the case had terminated favorably, but the instruments had been brought in and exhibited to the patient, and she was so indignant at my bringing them to the house, that I was dismissed from the case at once.

It is not the fashion to take obstetric instruments to the patient's house on the first visit, but most certainly *dignum et justum est* to do so.

What joy the physician brings into the house by saving the first child!

What remorse he causes by failure!

These considerations should overbalance all feelings of delicacy in a matter of this kind.

It is a well known fact that the head often remains behind in pelvic presentations, and the use of the forceps is required to save the child.

In an affair of life and death, feelings of delicacy should be laid aside, and then more children could be saved, and parents spared from much grief.—*Med. and Surg. Reporter.*

